# NetBID2 provides comprehensive hidden driver analysis

**DOI:** 10.1038/s41467-023-38335-6

**Published:** 2023-05-04

**Authors:** Xinran Dong, Liang Ding, Andrew Thrasher, Xinge Wang, Jingjing Liu, Qingfei Pan, Jordan Rash, Yogesh Dhungana, Xu Yang, Isabel Risch, Yuxin Li, Lei Yan, Michael Rusch, Clay McLeod, Koon-Kiu Yan, Junmin Peng, Hongbo Chi, Jinghui Zhang, Jiyang Yu

**Affiliations:** 1 Department of Computational Biology, St. Jude Children’s Research Hospital, Memphis, TN 38105 USA; 2 Graduate School of Biomedical Sciences, St. Jude Children’s Research Hospital, Memphis, TN 38105 USA; 3 Department of Immunology, St. Jude Children’s Research Hospital, Memphis, TN 38105 USA; 4 Departments of Structural Biology and Developmental Neurobiology, Centre for Proteomics and Metabolomics, St. Jude Children’s Research Hospital, Memphis, TN 38105 USA; 5 Present Address: Center for Molecular Medicine, Children’s Hospital of Fudan University, Shanghai, 201102 P.R. China; 6 Present Address: Department of Bioengineering, University of Illinois at Chicago, Chicago, IL 60607 USA

**Keywords:** Software, Systems analysis, Data integration, Reverse engineering, Bioinformatics

## Abstract

Many signaling and other genes known as “hidden” drivers may not be genetically or epigenetically altered or differentially expressed at the mRNA or protein levels, but, rather, drive a phenotype such as tumorigenesis via post-translational modification or other mechanisms. However, conventional approaches based on genomics or differential expression are limited in exposing such hidden drivers. Here, we present a comprehensive algorithm and toolkit NetBID2 (data-driven network-based Bayesian inference of drivers, version 2), which reverse-engineers context-specific interactomes and integrates network activity inferred from large-scale multi-omics data, empowering the identification of hidden drivers that could not be detected by traditional analyses. NetBID2 has substantially re-engineered the previous prototype version by providing versatile data visualization and sophisticated statistical analyses, which strongly facilitate researchers for result interpretation through end-to-end multi-omics data analysis. We demonstrate the power of NetBID2 using three hidden driver examples. We deploy NetBID2 Viewer, Runner, and Cloud apps with 145 context-specific gene regulatory and signaling networks across normal tissues and paediatric and adult cancers to facilitate end-to-end analysis, real-time interactive visualization and cloud-based data sharing. NetBID2 is freely available at https://jyyulab.github.io/NetBID.

## Abstract (web summary)

It’s challenging to capture “hidden” drivers that may not be genetically-altered or differentially-expressed from omics data. Here the authors developed NetBID2, a comprehensive network-based toolbox with versatile features, enabling the integration of multi-omics data to expose such hidden drivers.

## Introduction

Omics technologies, including next-generation sequencing, have played essential roles in identifying genetic/epigenetic alterations and abnormally expressed genes and proteins involved in homeostasis and diseases^1^. However, many signaling proteins (e.g., kinases), transcription factors, and other factors that are crucial drivers of phenotypes are not genetically/epigenetically altered or differentially expressed at the mRNA or protein level but are instead altered by post-translational or other modifications^2,3^; hence, they are termed hidden drivers. Conventional mutation analysis and differential expression analysis may not be able to capture them. Moreover, hidden drivers may operate in a context-dependent fashion, making them difficult to capture by knowledge-based pathway enrichment analysis.

Signaling hidden drivers are most likely druggable^4,5^, making them ideal therapeutic targets. Current targeted therapies against signaling drivers for cancer treatment rely primarily on gene mutations^6^; however, actionable mutations are present in less than 25% of human cancers^7^. The most frequently altered oncogenes and tumor suppressors, including MYC, KRAS, and TP53, are thus far undruggable^8^, and many patients carry no known cancer mutations. On the other hand, known genetics-based targeted therapeutics may target hidden drivers in a different cancer context that are not driven by genomic alternations. For example, dasatinib, a known ABL inhibitor, was approved to treat ALL with the BCR-ABL1 fusion or fusions involving other ABL class kinases^9–11^; however, a recent study showed that dasatinib is also effective in T-cell acute lymphoblastic leukemia (T-ALL) that has no ABL alterations and the network-based systems pharmacology analysis identified that LCK is the hidden driver of unexpected dasatinib sensitivity in T-ALL in a non-genetic-dependent manner^12^. Beyond genomics, network-inferred hidden drivers, especially signaling drivers, are potential therapeutic targets and are indispensable for precision cancer medicine.

Existing biomarkers of most targeted therapies, based on single-gene mutations or protein expression^6^, have limited predictive power. For example, more than 50% of patients with HER2 + breast cancer do not respond to anti-HER2 therapy^13^. Transcriptomics-based approaches showed promise in predicting in vivo and patient responses to anti-cancer therapies^14^. For example, a recent study showed that a network-based HDAC6 biomarker was able to predict preclinical and clinical responses to the HDAC6 inhibitor ricolinostat in breast cancer^15,16^. Integrative multi-gene-based companion diagnosis biomarkers, particularly network-based biomarkers, have massive potential to stratify patients for targeted therapies and immunotherapies.

**Fig. 1:**
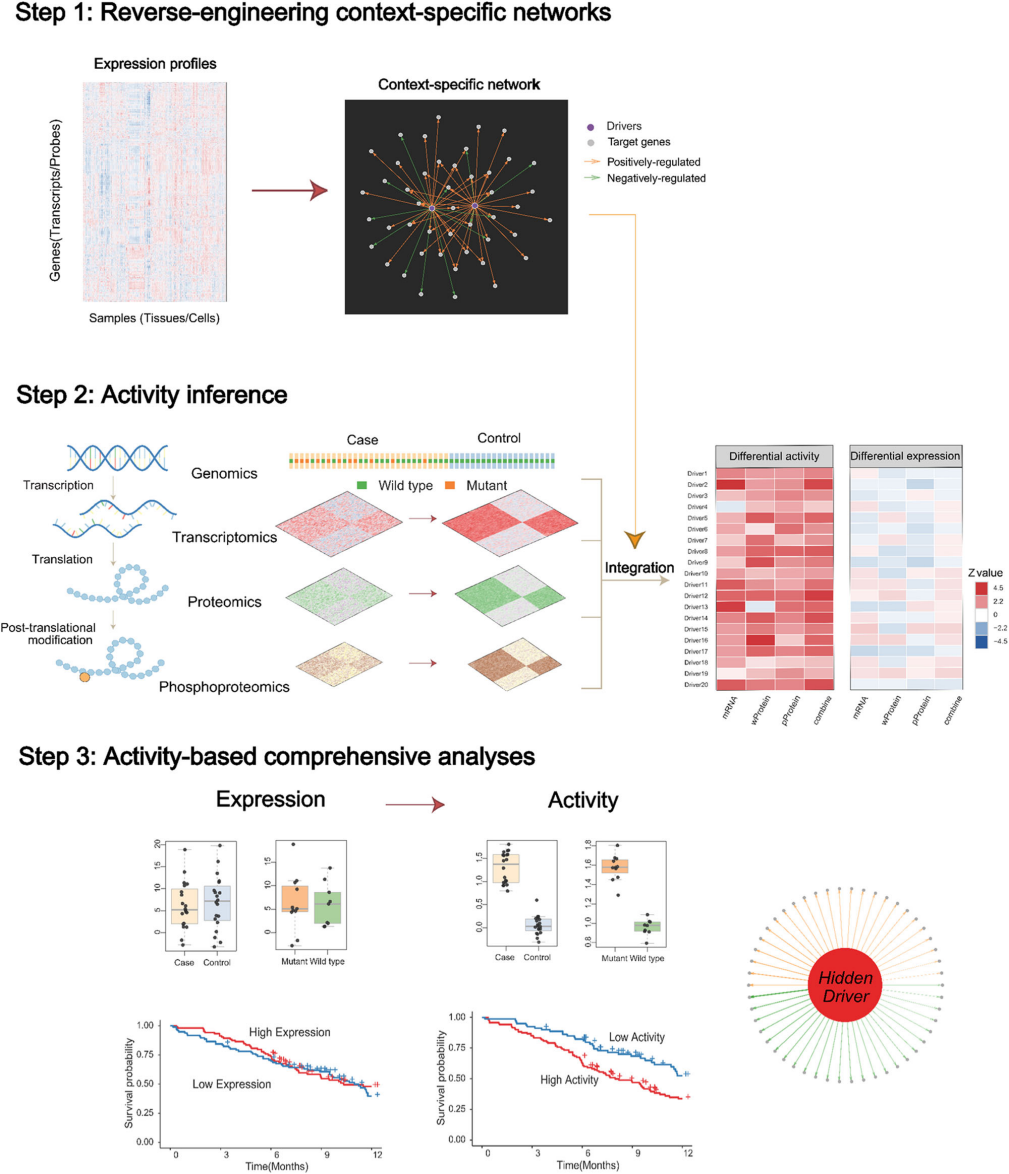
Overview of hidden driver analysis by NetBID2. Multi-omics data such as genomics, transcriptomics, proteomics, and phosphoproteomics, which measure the quantitative characteristics of genes at different stages, can be used to identify essential drivers by traditional differential analysis in case-control studies. However, many crucial drivers, especially “hidden drivers”, do not show much differential expression but still play an important role in biological processes of interest. NetBID2 has provided a comprehensive toolkit to explore the “hidden drivers” by the following key steps. Step 1: Reverse-engineering context-specific networks from a large-scale expression profile. Step 2: Activity inference from multi-omics data by summarizing the expression pattern of the candidate driver’s predicted target genes. Step 3: Do statistical analysis to find drivers with significantly differential activity and integrate for multi-omics. In previous applications, NetBID2 can successfully identify “hidden drivers” with significantly differential activity but no significant differential expression in case vs. control and mutant vs. wild-type comparison studies. Besides, the driver’s activity level rather than expression level shows better performance in survival probability analysis.

To expose such hidden drivers by using multi-omics data, we have developed a comprehensive data-driven, network-based algorithm and toolkit, NetBID2 (data-driven network-based Bayesian inference of drivers, version 2) (Fig. 1). In NetBID2, we have substantially re-engineered the prototype version of NetBID that has successfully identified MST1 as a hidden driver in selectively programming CD8α^+^ dendritic cells for anti-tumor immunity^17^, *CELSR2* as a negative driver of chemo-resistance in ALL^18^, *LCK* as a non-genetic driver of unexpected dasatinib sensitivity in T-ALL^12^, and *HDAC6* as a non-oncogene addition hub of subtypes of breast cancer^15^. To quantify the driver’s regulatory potential, the concept “activity” is defined to summarize the ability to control the expression of its transcriptional targets. Different from expression, the driver’s activity can be influenced not only by its RNA transcription but also by its protein synthesis, degradation, post-translational modification, complex formation, subcellular localization, and others. Hidden drivers usually exhibit differential activity instead of differential expression. NetBID prototype was a proof-of-concept version that has proven to be powerful in many successful applications.

Building on NetBID, we developed NetBID2, a comprehensive, versatile, and user-friendly software package of hidden driver inference toolkit, including state-of-the-art network analysis, gene expression analysis, functional analysis, meta-analysis, and visualizations. More specifically, NetBID2 provides functions and features for input data processing, normalization, batch correction, quality control of input datasets, and generated networks. It includes visualization features that facilitate users who might not have coding background. Further, the new driver activity inference considers the direction of targets. Therefore, NetBID2 has a variety of applications. For example, it can integrate cancer genomic data with transcriptomics and other omics data to capture hidden cancer drivers that may not show genomic alterations or differential expression.

## Results

### Key features of NetBID2

NetBID2 includes the following key features (Fig. S1): (1) *Reverse-engineering context-specific networks*. NetBID2 uses the latest version of SJARACNe for reverse-engineering networks from transcriptomics and proteomics data^19^. The SJARACNe uses the Common Workflow Language (CWL) to support multiple parallel computing platforms and improves the efficiency of network inference from large-scale data, including proteomics data and single-cell transcriptomics data. (2) *Activity inference*. NetBID2 introduces a “weighted mean” activity inference algorithm that summarizes the expression pattern of the target genes by taking into account both the strength and direction of the driver’s interaction with its predicted target genes. (3) *Visualization and comprehensive analyses*. NetBID2 provides versatile data visualization and sophisticated bioinformatics/statistical analyses, including differential expression analysis, gene set enrichment analysis (GSEA), network analysis, Bayesian analysis, and meta-analysis. These greatly facilitate the interpretation of results through end-to-end multi-omics data analysis. (4) *QC reporting*. NetBID2 implements state-of-the-art quality control HTML reporting of gene expression/activity and network data.

### Visualization and cloud apps of NetBID2

For the benefit of users with limited or no coding experience, we have developed the following interactive web and cloud applications of NetBID2:

1. *NetBID2 Viewer*. This interactive visualizer enables users to upload their NetBID2 output object and to explore the NetBID2 results interactively with an array of visualizations, including volcano plots, GSEA plots, heatmaps, functional enrichment plots, bubble plots, target networks, box plots, and driver target enrichment plots. An example of a live viewer is available at https://yulab-stjude.shinyapps.io/NetBID2_Viewer.
2. *NetBID2 Runner*. This enables users to perform the one-step NetBID2 hidden-driver analysis and generate a master table with a detailed R data file containing the project datasets. We developed a NetBIDshiny R package to produce the NetBID2 Viewer and Runner apps, and users can install them locally and/or publicly.
3. *NetBID2 Cloud App*. To exploit the power of cloud computing, we developed a cloud app for NetBID2 and deployed it on the NCI Cancer Genomics Cloud, which hosts the world’s largest cancer genomic datasets alongside thousands of bioinformatics tools.

### A resource of 145 context-specific gene regulatory and signaling networks

To facilitate the use of NetBID2 Runner, we have built a network resource of transcription factor and signaling protein networks for 145 normal and cancer contexts using our improved SJARACNe algorithm. Specifically, it includes 48 normal tissues from the Genotype Tissue Expression project (GTEx)^20^, 51 pediatric cancer types/subtypes from the Therapeutically Applicable Research to Generate Effective Treatments initiative (TARGET)^21^, and 46 adult cancer types/subtypes from The Cancer Genome Atlas (TCGA)^22^. It contains >145 million interactions in total. We have used NetBID2 to generate comprehensive QC reports for each of the 145 networks (Table S1), all of which have reasonable regulon sizes (Fig. S2) and scale-free features (Fig. S3). We also used the HALLMARK *MYC* targets to evaluate the *MYC* subnetworks in each of the normal tissues and cancer types, over half of which showed significant enrichment (Fig. S4).

### Example 1: NetBID2 identified *MYC* as a hidden driver in KRAS-driven LUAD

**Fig. 2:**
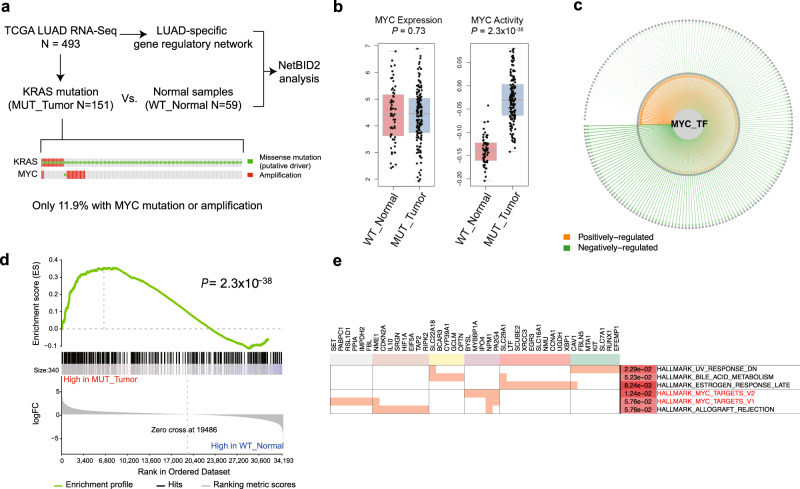
NetBID2 identifies *MYC* as a hidden driver for *KRAS*-mutant lung adenocarcinoma (LUAD). **a** Dataset and basic NetBID2 analysis workflow and oncoPrint of *KRAS* and *MYC* in LUAD from the TCGA data. **b** *MYC* activity inferred by NetBID2 (right) and its expression (left) in *KRAS*-mutant LUAD (*n* = 151 biologically independent samples) and wild-type normal samples (*n* = 59 biologically independent samples). The *P* values were estimated using two-tailed t test (getDE.limma.2G function in NetBID2). The center line represents the median and whiskers represents maximum (Q3 + 1.5*IQR) and minimum value (Q1 + 1.5*IQR). **c** The *MYC* transcription regulatory subnetwork in LUAD, inferred by SJARACNe from LUAD RNA-seq data. The orange and green edges correspond to positive and negative targets, respectively. The edge width is proportional to the mutual information of the interaction. **d** GSEA plot of the *MYC*-LUAD regulon for the differential expression in *KRAS*-mutant LUAD vs. wild-type normal samples. The *P* values were estimated using two-tailed t test (getDE.limma.2G function in NetBID2). **e** Function enrichment of the *MYC* target genes. Each box shaded in orange in the plot region indicates that the corresponding gene (column) is part of the pathway (row).

To demonstrate the power of NetBID2, we first present examples of hidden drivers in adult and pediatric cancers. The first example is *MYC* in *KRAS*-driven lung adenocarcinoma (LUAD). *MYC* was recognized as a functional driver of *KRAS*-mutant LUAD because *MYC* knockout could eradicate *KRAS*-driven lung cancer in mice^23^. However, conventional analysis of a TCGA LUAD cohort^24^ identified no significant association of *MYC* with *KRAS* mutation: only 11.9% of *KRAS*-mutant samples also harbored an *MYC* mutation or amplification (Fig. 2a), and *MYC* exhibited no differential expression in *KRAS*-driven LUAD vs. wild-type normal samples (*P* = 0.73) (Fig. 2b). In contrast, by using NetBID2, we could reconstruct a LUAD-specific interactome from 493 LUAD RNA-seq profiles and use the *MYC* subnetwork (Fig. 2c) to infer its protein activity, which significantly differentiated mutant *KRAS* from wild type (*P* = 2.3 × 10^−38^) (Fig. 2b, d). The power of NetBID2 to capture *MYC* as a hidden driver of *KRAS*-driven LUAD partially relies on the data-driven *MYC* regulon (Fig. 2c), reflecting both its known functions and unreported ones in LUAD (Fig. 2e). The predicted *MYC* targets in LUAD were also validated by ChIP-seq analysis of A549, a LUAD cell line, from the ENCODE project^25^ and footprinting analysis^26^ of the A549 ATAC-seq data in ENCODE (Fig. S5a, b). In addition to *MYC*, we also used the A549 ATAC-seq data to evaluate the overall LUAD TF network, in which all TFs showed significant enrichment between SJARACNe-predicted targets and the targets defined by A549 ATAC-seq analysis (Fig. S5c).

### Example 2: NetBID2 identified *NOTCH1* as a hidden driver in T-ALL

**Fig. 3:**
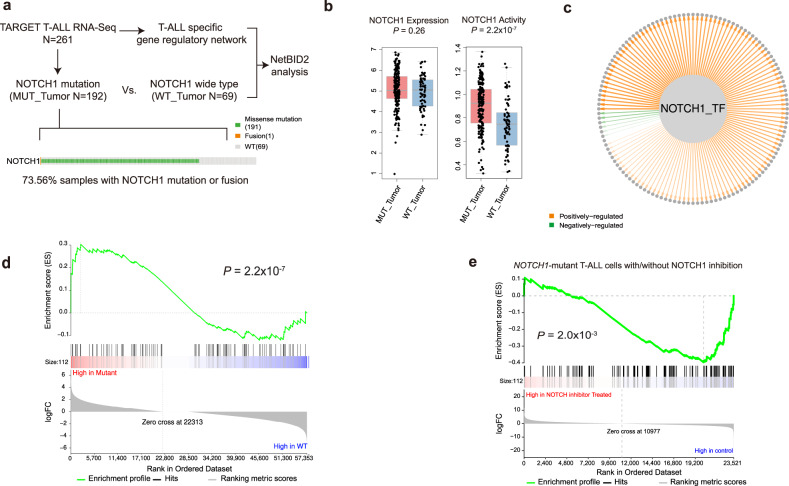
NetBID2 captures NOTCH1 protein activity in *NOTCH1*-mutant T-cell acute lymphoblastic leukemia (T-ALL). **a** Dataset and basic NetBID2 analysis workflow and oncoPrint of *NOTCH1* in T-ALL from the TARGET data. **b** *NOTCH1* activity inferred by NetBID2 (right) and expression (left) in *NOTCH1*-mutant (*n* = 192 biologically independent samples) and wild-type T-ALL samples (*n* = 69 biologically independent samples). The *P* values were estimated using two-tailed t test (getDE.limma.2G function in NetBID2). The center line represents the median and whiskers represents maximum (Q3 + 1.5*IQR) and minimum value (Q1 + 1.5*IQR). **c** The *NOTCH1* transcription regulatory subnetwork in T-ALL, as inferred by SJARACNe from T-ALL RNA-seq data. Orange and green edges correspond to positive and negative targets, respectively. The edge width is proportional to the mutual information of the interaction. **d** GSEA plot of the *NOTCH1* regulon for the differential expression of *NOTCH1*-mutant vs. wild-type T-ALL samples. **e** GSEA plot of the *NOTCH1* regulon in the differential expression of *NOTCH1*-mutant T-ALL cells with or without *NOTCH1* inhibition by a gamma-secretase inhibitor (GSE6495)^28^. **d**, **e** The *P* values were estimated using two-tailed t test (getDE.limma.2G function in NetBID2).

The second example is *NOTCH1*, the primary oncogene that is mutated in approximately 74% of childhood T-ALL, based on a recent analysis of the TARGET RNA-seq data^27^ (Fig. 3a). However, *NOTCH*1 showed no differential expression in mutant vs. wild-type T-ALL samples (*P* = 0.26) (Fig. 3b). In contrast, by using NetBID2, we could reconstruct a T-ALL-specific interactome from RNA-seq profiles of T-ALL primary samples (*N* = 261) and use the *NOTCH*1 subnetwork (Fig. 3c) to infer its protein activity, which significantly differentiated mutant cases from wild-type cases *(P* = 2.2 × 10^−7^) (Fig. 3b, d). The *NOTCH*1 regulon (Fig. 3c) inferred from T-ALL RNA-seq profiles is significantly enriched by its putative targets, defined by differentially expressed genes in *NOTCH*1-mutant T-ALL cells with and without *NOTCH*1 inhibition^28^ (Fig. 3e). These results further established the power of NetBID2 to capture protein activity by using a context-specific network.

### Example 3: NetBID2 identified *Gabpa* as a hidden driver in CD4^+^ T cells upon TCR stimulation

**Fig. 4:**
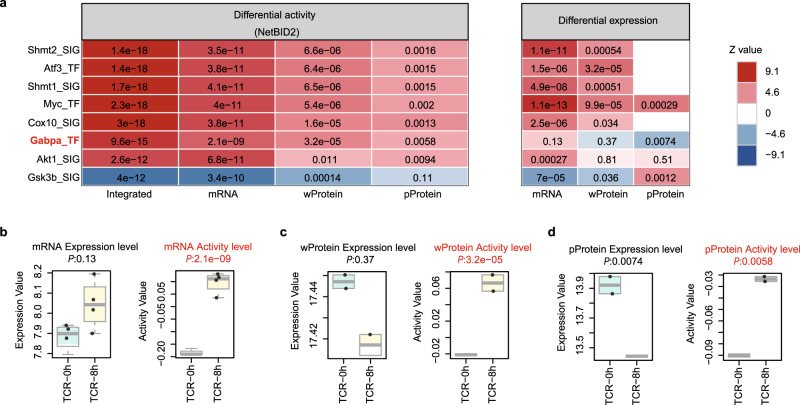
NetBID2 identifies *Gabpa* as a hidden driver in T-cell receptor (TCR) stimulation from 0 h to 8 h. **a** Eight drivers that are differentially activated in TCR stimulation from 0 h to 8 h as inferred by NetBID2. Left, the NetBID2 panel indicates the significance level (color coded by z score; labeled values are adjusted *P* values) in integrated analysis, transcriptomics (mRNA) data, whole-proteomics (wProtein) data, and phosphoproteomics (pProtein) data. Right, differential expression of the drivers (color coded by z score; labeled values are adjusted *P* values). TF: transcription factor; SIG: signaling factor. **b** *Gabpa* activity inferred by NetBID2 at the mRNA level (right) and the original mRNA expression (left) in TCR-8h (*n* = 4 biologically independent samples) and TCR-0h samples (*n* = 4 biologically independent samples). **c** *Gabpa* activity inferred by NetBID2 at the wProtein level (right) and the original wProtein expression (left) in TCR-8h (*n* = 2 biologically independent samples) and TCR-0h samples (*n* = 2 biologically independent samples). **d** *Gabpa* activity inferred by NetBID2 at the pProtein level (right) and the original pProtein expression (left) in TCR-8h (*n* = 2 biologically independent samples) and TCR-0h samples (*n* = 2 biologically independent samples). In **a**–**d**, the *P* values were estimated using two-tailed t test (getDE.limma.2G function in NetBID2). The central line represents the median and whiskers represents maximum (Q3 + 1.5*IQR) and minimum value (Q1 + 1.5*IQR).

We present one more example in which transcriptomics (mRNA), whole proteomics (wProtein), and phosphoproteomics (pProtein) data were integrated to capture hidden drivers of the naive CD4^+^ T-cell response upon T-cell receptor (TCR) stimulation. We collected bulk transcriptomics, whole-proteomics, and phosphoproteomics data for CD4^+^ T cells before and after TCR stimulation in two previous studies^29,30^. Using NetBID2, we reconstructed a naive CD4^+^ T-cell-specific gene–gene interaction network from the transcriptomic profiles of 24 CD4^+^ T-cell samples and integrated different levels of omics data to identify drivers in response to TCR stimulation at 8 h vs. 0 h. To evaluate the performance of NetBID2, we curated eight positive control drivers (*Cox10*, *Shmt1*, *Shmt2*, *Myc*, *Atf3*, *Gabpa*, *Akt1*, and *Gsk3b*) that had previously been identified with experimental validations^31–35^ (Table S2). Remarkably, NetBID2 could identify all of them (Fig. 4a) (with adjusted *P* < 4.0 × 10^−12^), with the transcription factor *Gabpa* being revealed as a particularly notable hidden driver (Fig. 4b–d). *Gabpa* is a functionally validated positive driver of T-cell homeostasis and immunity^33,34^, but its mRNA and whole-protein expression showed no significant change, and the phosphoprotein expression was even down-regulated after 8 h of TCR stimulation. However, NetBID2 was able to capture its up-regulated activity at all three levels, namely mRNA, wProtein, and pProtein.

This example provided us an opportunity to evaluate the effects of different omics modalities and input sample sizes on hidden driver inference. We compared the statistics using each modality alone, mRNA + wProtein, and all three (Fig. S6). First, mRNA alone consistently produced better statistical significance than wProtein alone in all 8 cases, and wProtein alone is better than pProtein alone in all except *Akt1*. Second, all three omics modalities produced better statistical significance than each alone in all 8 cases. Third, combining mRNA, wProtein, and pProtein had similar performance as compared to the former two alone, suggesting that the contribution of pProtein is rather mild. We also systematically examined the overall correlations of NetBID2 z-statistics using all three omics data using each of them alone (Fig. S7). The results suggested that mRNA and wProtein had similar correlations with integrated, with a correlation coefficient of 0.928 and 0.918, respectively. The pProtein alone had a worse correlation than mRNA and wProtein, likely due to the noise and limited information (e.g., phosphorylation only) of phosphoproteomics data. We further tested the model performance by gradually increasing the number of samples used for network construction, and NetBID2 performance was improved upon the increase in sample size (Fig. S8). In summary, proteomics, especially whole proteomics data when available and large input sample size, will greatly enhance the hidden driver discovery by NetBID2.

### “Weighted mean” outperforms “mean” for protein activity inference

**Fig. 5:**
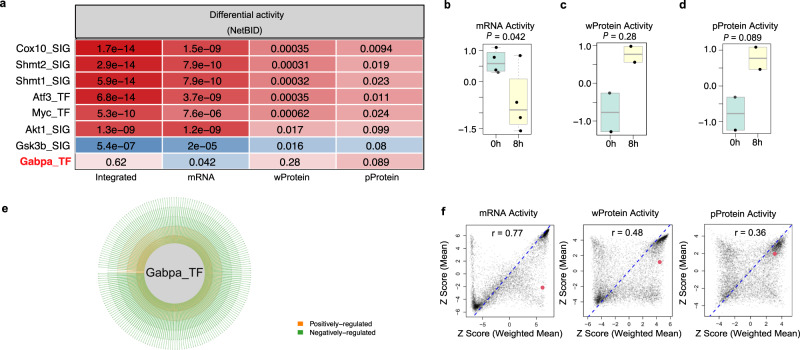
Comparison of the “weighted mean” method in NetBID2 with the “mean” approach in the NetBID prototype to infer driver activities in the case of TCR stimulation from 0 h to 8 h. **a** Differential activity statistics of the eight positive control drivers of CD4^+^ T-cell activation were obtained using the “mean” approach to infer activity in integrated data and at the mRNA, wProtein, and pProtein levels. TF: transcription factor; SIG: signaling factor. **b**–**d** *Gabpa* activity inferred by the “mean” approach showed no significant difference between TCR-8h and TCR-0h samples at the mRNA (*n* = 4 biologically independent samples for each group), wProtein (*n* = 2 biologically independent samples for each group), and pProtein levels (*n* = 2 biologically independent samples for each group). The *P* values were estimated using two-tailed t test (getDE.limma.2G function in NetBID2). The center line represents the median and whiskers represents maximum (Q3 + 1.5*IQR) and minimum value (Q1 + 1.5*IQR). **e** The *Gabpa* transcription regulatory subnetwork in CD4^+^ T cells as inferred by SJARACNe from RNA-seq profiles of CD4^+^ T cells. The orange and green edges correspond to positive and negative targets, respectively. The edge width is proportional to the mutual information of the interaction. **f** The correlation of z scores for differential activity, comparing TCR-8h with TCR-0h samples by using the “weighted mean” and “mean” approaches to infer driver activity from mRNA, wProtein, and pProtein data. The Pearson correlation coefficients (r) are labeled inside the box. The red points represent *Gabpa*.

We also used the TCR response example with mRNA, wProtein, and pProtein data to evaluate the “weighted mean” method for activity inference in NetBID2 by comparing it with the “mean” approach used in the NetBID prototype. Notably, the “weighted mean” approach (Fig. 4a) yielded stronger statistical evidence of differential activity than did the “mean” based method (Fig. 5a) for all eight positive control drivers. In particular, the “mean” approach failed to identify *Akt1* (*P* = 0.099) and *Gsk3b* (*P* = 0.08) at the pProtein level and *Gabpa* at the mRNA (*P* = 0.042, but wrong direction), wProtein (*P* = 0.28), and pProtein (*P* = 0.089) levels (Fig. 5b–e). Overall, the differential activity scores derived with the “weighted mean” and “mean” approaches correlated positively with each other, although the correlation at the mRNA level (Pearson correlation coefficient *r* = 0.77) is much stronger than at the wProtein (*r* = 0.48) and pProtein (*r* = 0.36) levels (Fig. 5f). However, the “weighted mean” outperformed “mean” in inferring driver activity and nominating hidden drivers such as *Gabpa* that was completely missed by “mean” based approach.

## Discussion

We have demonstrated that NetBID2 goes beyond genomics mutation and conventional differential expression to infer protein activity from data-driven and context-specific networks, thereby exposing hidden drivers of various biological processes. NetBID2 can integrate multiple omics data, including transcriptomics, proteomics, and phosphoproteomics, which is different from existing gene-expression-focused approaches such as VIPER^36^. NetBID2 can infer interaction networks and activities of not only transcription factors (TFs) but also signaling proteins such as kinases, epigenetic modulators, metabolic factors, etc. We have demonstrated significant enrichment of NetBID2-inferred TF regulons with targets defined by TF ChIP-seq or ATAC-seq data from the matched contexts by using motif enrichment and footprinting analyses. NetBID2 identifies the downstream targets influenced by the hidden driver, but some targets could potentially be indirect targets and therefore cannot be discovered by ChIP-seq or ATAC-seq data. The TF networks can be further improved by integrating with TF ChIP-seq data or ATAC-seq data. The signaling networks can also be further improved by integration with protein–protein interaction networks reconstructed by affinity purification−mass spectrometry in specific contexts such as breast cancer^37^.

It is important to emphasize that the main goal of NetBID2 is to infer hidden drivers. Based on our activity framework, by aggregating the signal from a set of target genes, we can infer the role of the driver with stringent statistics. Nevertheless, the signal of individual targets could be weak, and thus individual targets could be viewed as secondary results.

Transcriptomics is still the primary input dataset for NetBID2 to uncover hidden drivers in most cases because of the limitations of proteomics and phosphoproteomics data. Despite the increasing number of proteomics profiles, it is still rarely available, especially phosphoproteomics data, compared to RNA-seq. Even when they are available, the sample size of proteomics data is usually small. Further, the latest TMT mass spectrum can detect >14,000 proteins^38^, but the coverage is still limited, given the intrinsic technical limitations. The batch effects of proteomics data make it even more challenging to analyze.

One limitation of NetBID2 is that it requires a relatively large sample-sized transcriptomics dataset with the same biological condition as the dataset used for driver inference to reconstruct a context-matched network. In some cases, it might be challenging to find matched datasets. The single-cell transcriptomics may solve the sample size issue. NetBID2 has the potential to be applied to single-cell transcriptomics data for cell-type-specific networks and hidden driver inference. The intrinsic sparseness of single-cell RNA-seq data will make the network reconstruction challenging. Re-engineering SJARACNe and pseudo-bulking or meta-cell analysis will be needed to overcome the dropout effects for reasonable gene–gene correlation estimation from single-cell data. The availability of matched scATAC-seq data will help improve the TF network reverse-engineering. The network-inferred activity profiles at the single-cell level will be able to rescue the detection of many genes with many zero counts in single-cell expression data. The less-sparse single-cell activity map may further improve the clustering and integration analysis of single-cell data from different cohorts.

Another limitation of NetBID2 is that the activity inference currently focuses on TF and SIG drivers and assumes the correlation of the driver’s activity with its expression. This strategy may miss some drivers that do not function as TF or SIG or whose activities are independent of expression. A potential solution might be using the first-neighbor genes to infer the activity of any given gene since our data-driven networks cover the whole transcriptome space. However, further evaluation of new activity inference and non-TF/SIG drivers will be required.

In summary, NetBID2 is a powerful and comprehensive tool to integrate with which multi-omics data and nominate hidden drivers in cancer and other biological conditions that conventional mutation, differential expression, and pathway analyses may fail to identify. This tool will benefit researchers in the post-omics era, enabling them to identify non-genetic dependencies and therapeutic targets for cancer and other diseases. The NetBID2 Viewer, Runner, and Cloud apps, with a valuable resource of 145 data-driven and context-specific networks, will facilitate the broad and reproducible use of NetBID2 with enhanced visualization, data management, and results sharing.

## Methods

### Input datasets for NetBID2

The two required input datasets of NetBID2 include (1) a transcriptomic dataset in the relevant biological condition used for network construction and (2) an expression profiling dataset (at least one omics modality from RNA-seq, whole proteomics, and phosphoproteomics) with experimental design (e.g., case vs. control, phenotype groups) for driver inference. For the network construction, the input sample size is recommended to be >20, which is enough for generating reproducible networks. Although there is no optimal one-size-fits-all solution in practice, we normally recommend a few hundred if possible and perform the QC of the input expression data carefully. For the driver inference expression dataset, NetBID2 does not require all three modalities (transcriptomics, proteomics, phosphoproteomics)—at least one modality omics dataset will be sufficient. The transcriptomics data that cover genome-wide gene expression levels outperforms other modalities generally and the integration of proteomics will increase the power of hidden driver inference.

### A typical workflow of NetBID2

When the input is prepared, a typical workflow of NetBID2 includes the following. (1) Perform QC for input gene expression profiles of network inference and driver inference. (2) Reconstruct context-specific TF and SIG networks, respectively, with the input of transcriptomic profiles and curated TF and SIG driver lists by SJARACNe and perform network QC. (3) Calculate activity for candidate drivers in each of the driver inference datasets based on the SJARACNe-inferred TF and SIG networks. (4) Perform differential activity (DA) analysis for candidate drivers and differential expression (DE) analysis by BID (Bayesian inference of drivers). (5) Integrate DA and DE results using BID if more than one modality omics dataset is provided or more than one comparison is conducted. (6) Generate result objects (can be used as input for NetBID2 Viewer), master tables, and all kinds of visualization plots for top drivers or a driver of interest. (7) Perform functional enrichment analysis of top drivers with visualizations. A detailed step-by-step tutorial with an example and codes is described in the NetBID2 online.

### Processing and QC of input data

NetBID2 provides a series of functions with visualizations to process and QC different types of input datasets (e.g., microarray, RNA-seq, proteomics), including gene filtering, normalization, ID conversion, transcript-level to gene-level conversion, missing data imputation, outlier detection, dataset combination, batch effect detection, and removal, etc. The HTML QC report includes heatmap, PCA/MDS/UMAP plots, sample correlation plots, distribution plots, etc.

### Network reconstruction

With the transcriptomics data passing QC, NetBID2 prepares the input files for SJARACNe. SJARACNe uses the common workflow language (CWL) and node.js. It can be run on local machines and high-performance computing clusters. Conda virtual environment is recommended to set up the required Python and dependencies. Recommended and default parameters include the number of bootstraps (*n*) to be 100 and the consensus *p*-value (pc) to be 1e−5.

### Network QC

NetBID2 provides a detailed QC report for SJARACNe-inferred networks, including the following:

- Network overview properties. A table of basic statistics to characterize the network, including size and different centrality metrics (e.g., density, degree, eigenvector, PageRank, etc.).
- Individual driver subnetwork statistics. A table of detailed statistics for each individual driver subnetworks.
- Target size plot. A density over the histogram shows the distribution of nodes’ degree and the drivers’ target size. Our experience suggests that an average target size of around several hundred may be preferable.
- Scale-free check. The scale-free attribute is often used as a metric to check the robustness of a network. The R^2^ from the linear fitting between the degree (k) and degree distribution (pk) is used as the metric—the higher R^2^ is, the more scale-free and robust the network is.

An example of network a QC report can be found at: https://jyyulab.github.io/NetBID_shiny/docs/tutorial4online/TCGA_network_QC/LUAD.T_35321_16788_493netQC.html. QC reports for all 145 networks are available at https://jyyulab.github.io/NetBID_shiny.

### Activity inference

NetBID2 uses the “weighted mean” approach to calculate driver activity (cal.Activity function). It also provides other options, including “mean”, “maxmean”, and “absmean”. “Weighted mean” is the MI (mutual information) value with the sign of the Spearman correlation. For example, if the user chooses “weighted mean” to calculate the activity of a driver, then the higher the expression value of its positively regulated genes and the lower the expression value of its negatively regulated genes, the higher the activity value of that driver will be. Z-transformation to the expression matrix (std=TRUE in cal.activity function) is performed by default before calculating the activity. NetBID2 also generates QC reports for the activity matrix.

### Differential activity and differential expression analysis

NetBID2 uses Bayesian linear regression or BID approach for DA and DE analysis of two group comparisons by default. The default method of BID is “Bayesian”, but “MLE” is an alternative. For phenotypes with more than two groups, NetBID2 provides bid and limma functions.

### Integration of multiple DA/DE results

NetBID2 uses Stouffer’s method to combine statistics from multiple DA or DE results. It also provides Fisher’s approach to combine *p*-values only. For DE combination, NetBID2 also provides a combination of other statistics, including logFC, AveExpr, etc.

### Functional enrichment analysis

NetBID2 provides functions for comprehensive enrichment analysis and visualization. It supports different kinds of enrichment algorithms, including Fisher’s exact test, GSEA-like, two-set GSEA, activity-based enrichment, etc. It also provides biclustering analysis and plots (heatmap, bubble) of genes-pathways. Demos can be found on the NetBID2 tutorial: https://jyyulab.github.io/NetBID/docs/advanced_analysis.

### The resource of 145 prebuilt data-driven networks of normal tissues and cancers in NetBID2 Runner

In NetBID2 Runner for hidden driver analysis, we have prebuilt paired networks (a transcription factor network and a signaling network) of 48 normal tissues from GTEx^20^, 51 pediatric cancer types or subtypes from TARGET^21^, and 46 adult cancer types or subtypes from TCGA^22^ by using the SJARACNe^19^ algorithm with the default settings. The total number of interactions is >145 million. Detailed statistics and QC reports for each network are available in Table S1 or at https://jyyulab.github.io/NetBID_shiny.

### Statistics and reproducibility

No statistical method was used to predetermine sample size. In Example 1, LUAD-specific interactome were reconstructed from 493 LUAD RNA-seq profiles by SJARACNe. The activity level for all genes were inferred by NetBID2 cal.Activity function. The differential expression and activity analyses for genes between 151 tumor samples with KRAS mutation (MU_Tumor) and 59 normal samples (WT_Normal) were conducted by NetBID2 getDE.limma.2G function. The target genes for MYC_TF were extracted from the reconstructed network and the function enrichment analysis was performed by NetBID2 funcEnrich.Fisher function, visualized by draw.funcEnrich.cluster function. The GSEA plot was created by NetBID2 draw.GSEA function. Narrow Peaks for A549 Chip-Seq of MYC were downloaded from ENCODE (ENCFF542GMN) and annotated to hg38 known gene region by ChIPseeker with default settings. A549 ATAC-seq results were downloaded from ENCODE (ENCFF143XED) and annotated to hg38 known gene region by ChIPseeker with default settings. Footprinting analysis was performed to define MYC targets from ATAC-seq data. In Example 2, T-ALL-specific interactome were reconstructed from 261 T-ALL primary samples by SJARACNe. The activity level for all genes were inferred by NetBID2 cal.Activity function. The differential expression and activity analyses for genes between 192 tumor samples with NOTCH1 mutation (MUT_Tumor) and 69 without NOTCH1 mutation (WT_Tumor) were conducted by NetBID2 getDE.limma.2G function. The target genes for NOTCH1_TF were extracted from the reconstructed network. The expression from GSE6495 was processed by NetBID2 load.exp.GEO function and the differential expression profile was calculated by getDE.limma.2G function. The GSEA plot was created by NetBID2 draw.GSEA function. In Example 3, naive CD4^+^ T-cell-specific gene–gene interaction networks were reconstructed from the transcriptomic profiles of 24 CD4^+^ T-cell samples by SJARACNe. The activity level for all genes were inferred by NetBID2 cal.Activity function. The differential expression and activity analyses for genes between TCR stimulation at 8 h vs. 0 h were conducted by NetBID2 getDE.limma.2G function. The target genes for Gabpa_TF were extracted from the constructed network.

### Reporting summary

Further information on research design is available in the Nature Portfolio Reporting Summary linked to this article.

## Supplementary information

Supplementary Information

Reporting Summary

## Data Availability

The RNA-seq dataset for the non-small cell lung cancer are available at https://portal.gdc.cancer.gov/projects/TCGA-LUAD. The RNA-seq dataset for the T-cell acute lymphoblastic leukemia is available on https://platform.stjude.cloud/ and in the GEO database under the access code GSE6495. The whole-proteomics and phosphoproteomics datasets for T-cell activation is available in the Supplemental Data S1A and S1B of Tan et al.^30^, and the matched microarray dataset is available in the GEO database under the access code GSE51668^29^. A549 MYC ChIP and ATAC-seq data were downloaded from the ENCODE database^25^ under the access codes ENCFF542GMN and ENCFF143XED. Processed data is also available on Zenodo^39^. All other relevant data supporting the key findings of this study are available within the article and its Supplementary Information files. Source data are provided with this paper.

## Source data

Source data

## Code availability

The source code for NetBID2 is available as Supplementary Software and online at GitHub: https://github.com/jyyulab/NetBID and Zenodo^40^. The documentation with a tutorial is available online at https://jyyulab.github.io/NetBID. The NetBID prototype is available at https://github.com/jyyulab/NetBID/releases/tag/1.0.0. The new version of SJARACNe with CWL is available at https://github.com/jyyulab/SJARACNe. NetBIDshiny is available at https://github.com/jyyulab/NetBID_shiny and Zenodo^41^. The documentation with a tutorial is available online at https://jyyulab.github.io/NetBID_shiny. The NetBID2 Viewer and Runner demo apps generated by NetBIDshiny are available at https://yulab-stjude.shinyapps.io/NetBID2_Viewer and https://yulab-stjude.shinyapps.io/NetBID2_Runner, respectively. The NCI Cancer Genomics Cloud NetBID2 app is available at https://cgc.sbgenomics.com/public/apps/stjude/netbid/netbid.
