# Supplementary material for: NetBID2 provides comprehensive hidden driver analysis
Source: Nat Commun. 2023 May 4;14:2581. doi: 10.1038/s41467-023-38335-6 (PMC10160099; doi:10.1038/s41467-023-38335-6)
Supplement: Supplementary file 1 — Supplementary Information [file 41467_2023_38335_MOESM1_ESM.pdf]

Supplementary Information to

NetBID2 provides comprehensive hidden driver analysis

Dong, et al.

Supplementary Figures 1-8.

Supplementary Tables 1-2.

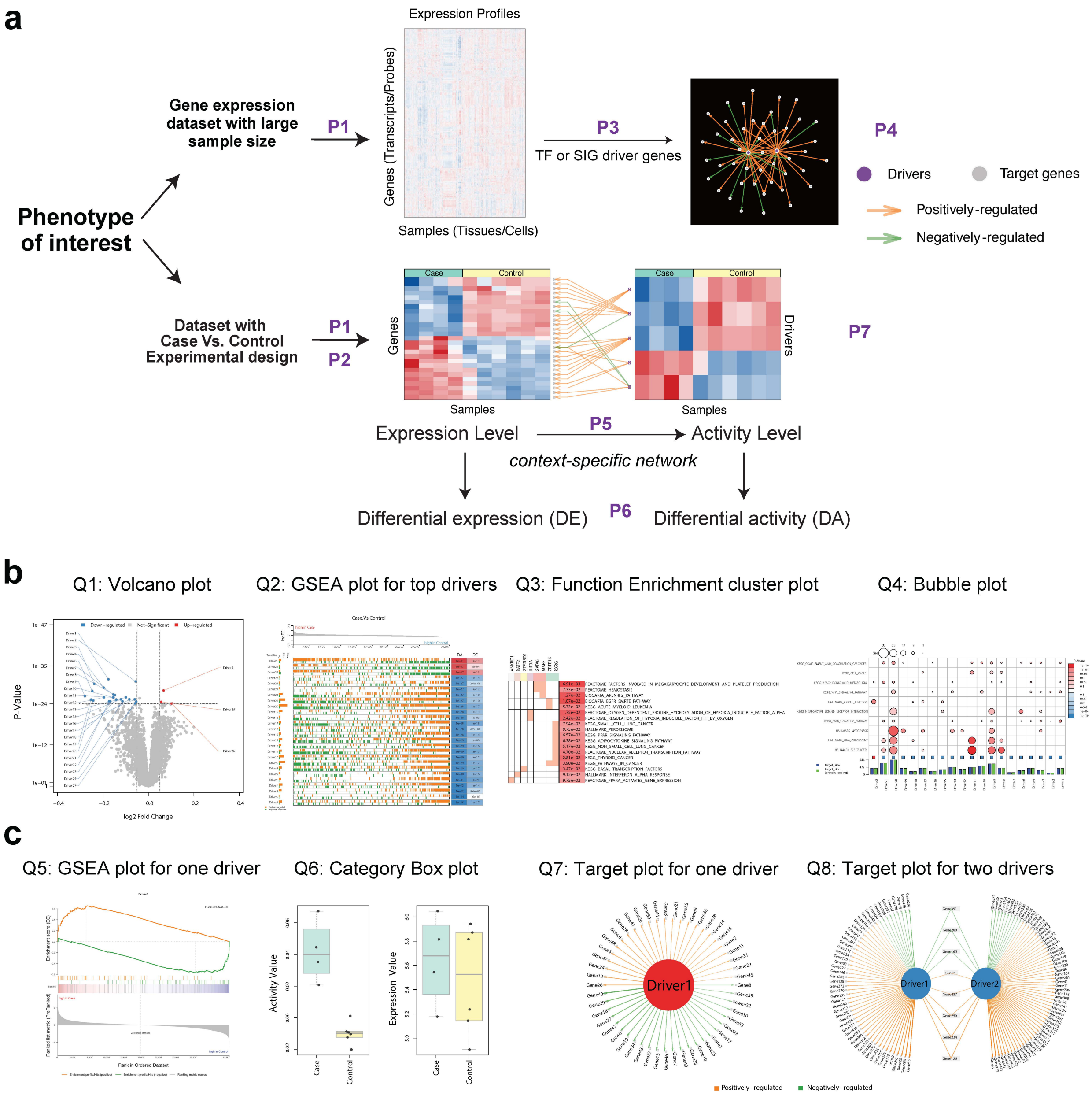

Figure S1. Key features and functions of NetBID2, making it a handy, comprehensive and practicable software for "hidden driver" analysis. (**a**) Key data processing functions include: P1: expression matrix pre-processing and quality assessment; P2: Gene ID Conversion (optional); P3: Context-specific gene regulatory network generation by SJARACNe; P4: Quality assessment for the network; P5: Activity calculation of drivers and gene sets; P6: Discovery of differentially expressed genes and differentially activated drivers; P7: Master table generation (excel/RData file) for drivers. (**b**) Key visualization functions for display of top drivers to answer: Q1: How to get the top drivers? Q2: How to interpret the significance of top drivers? Q3: What are the biological functions of these top drivers? Q4: What are the biological functions of the target genes of the top drivers? (**c**) Key visualization functions for display of selected drivers to answer: Q5: How to interpret the significance of the selected drivers? Q6: What is the expression/activity of this selected driver across subtypes of sample? Q7: How to visualize the network structure of the selected driver? Q8: How to visualize the network structure of two

selected drivers? More functions and demonstrations can be found in NetBID2 online tutorial and manual at https://jyyulab.github.io/NetBID.

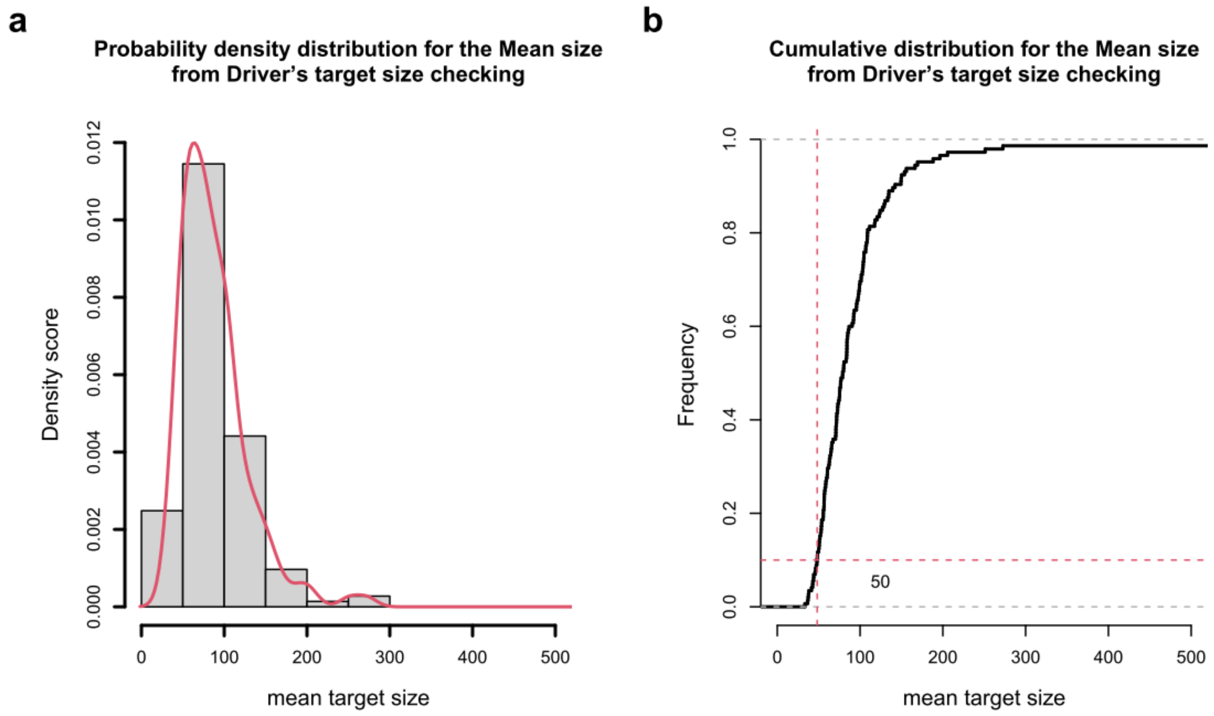

Figure S2. Summary of mean target size of 145 pre-built networks. The mean value for the driver's target size from each network was calculated. In total 145 networks were analyzed and the mean value distribution in five value intervals was summarized with a probability density plot (**a**) and cumulative distribution plot (**b**). 90% of 145 networks have a mean target size of more than 50. The overall mean is 101.

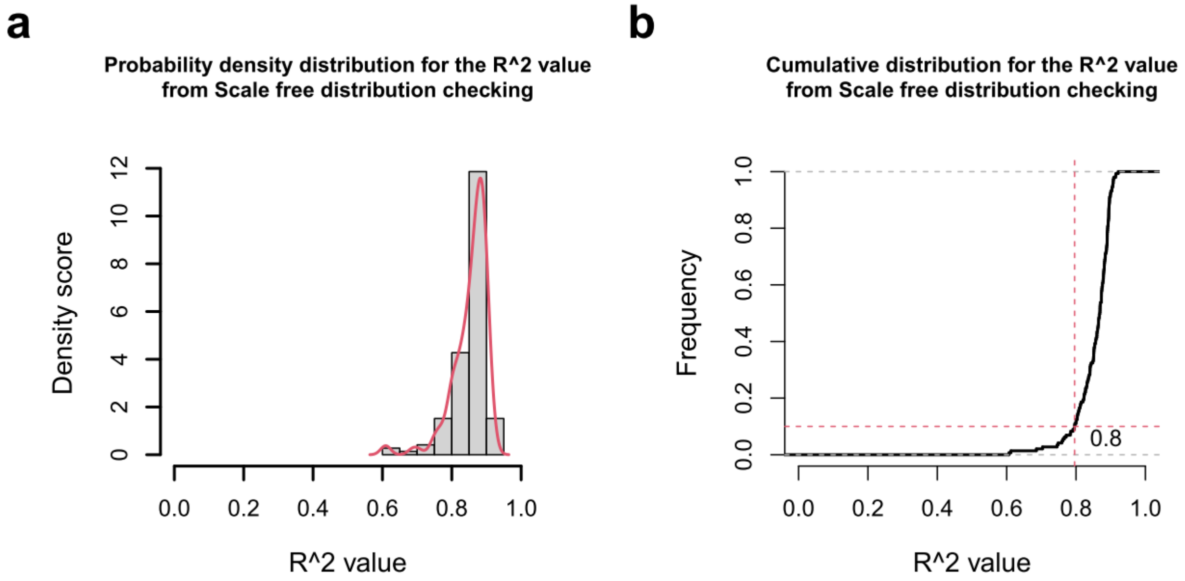

Figure S3. Scale-free check of 145 pre-built networks. For each network, the R^2 from the linear fitting between the degree (k) and degree distribution (pk) was calculated. In total, 145 networks were analyzed, and the R^2 value distribution with a probability density plot (**a**) and cumulative distribution plot (**b**). The overall mean is 0.86. 90% of our networks have an R^2 value higher than 0.8.

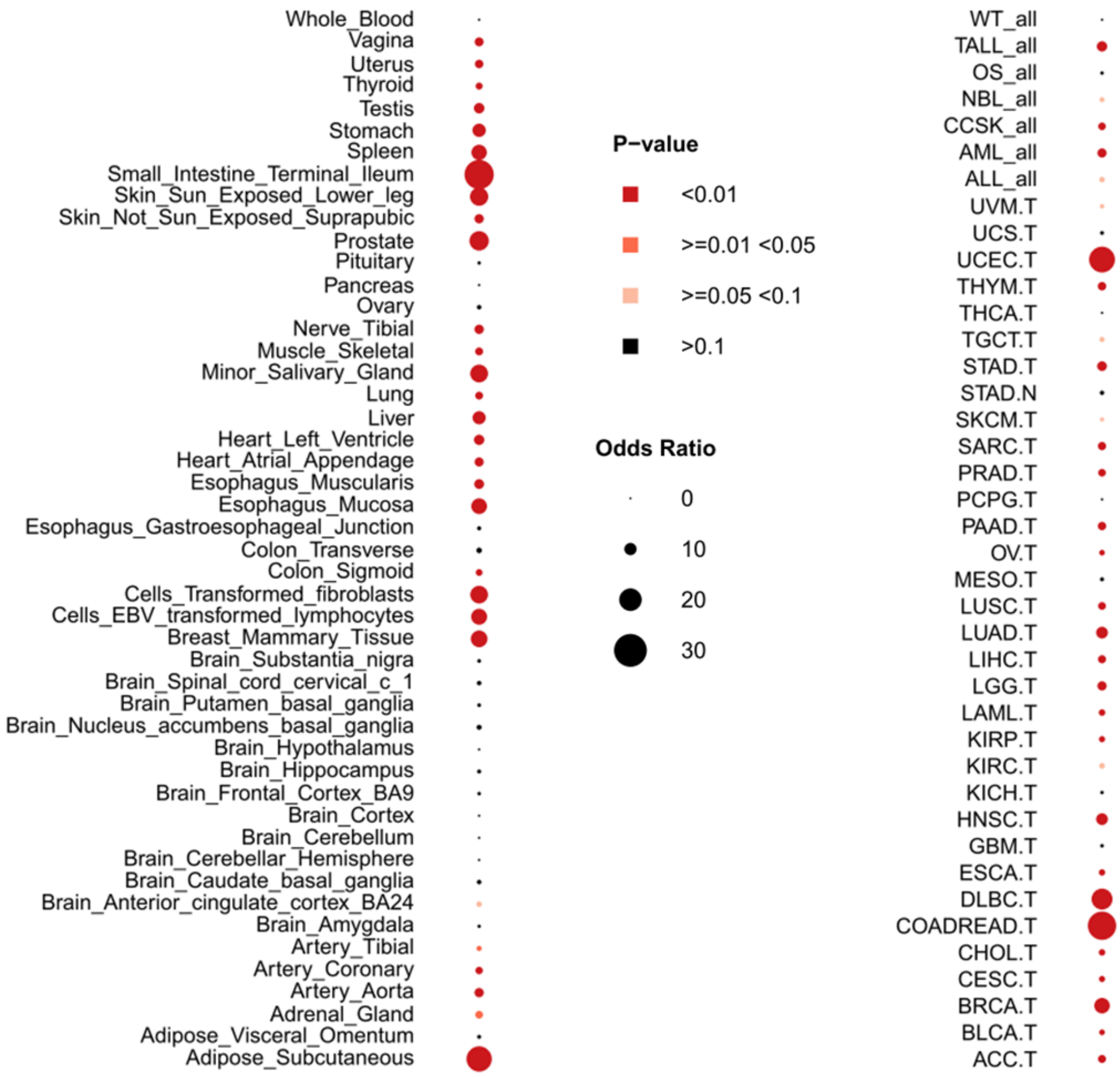

Figure S4. Validation of MYC subnetworks in normal tissues and different cancer types. Enrichment statistics for the SJARACNe-inferred MYC targets in different normal and cancer types with HALLMARK_MYC_TARGETS (the union of two MSigDB terms, HALLMARK_MYC_TARGETS_V1 and HALLMARK_MYC_TARGETS_V2). The color of each point represents the p-value for Fisher's Exact Test (one-sided), and the size is proportional to the odds ratio. For example, 28 out of 48 normal tissue (GTEx) networks (left) and 25 out of 40 cancer (TCGA and TARGET) networks showed significant enrichment ($p < 0.05$) of predicted MYC regulon with HALLMARK_MYC_TARGETS.

a

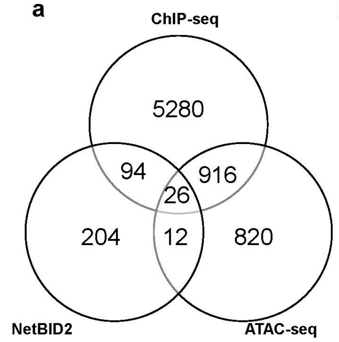

b

Target Set 1	Target Set 2	Set 1 Size	Set 2 Size	Overlap	Enrichment P-value	Enrichment Odds Ratio
NetBID2	ChIP-seq	336	6316	120	2.98e-37	5.11
NetBID2	ATAC-seq	336	1774	38	4.31e-13	4.51
NetBID2	ChIP-seq ∩ ATAC-seq	336	942	26	1.18e-11	5.70
ChIP-seq	ATAC-seq	6316	1774	942	<2e-16	11.9

C

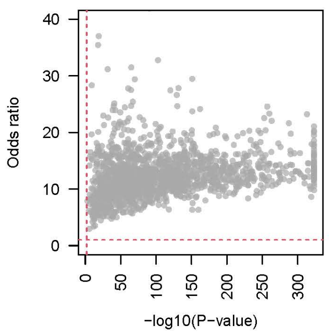

Figure S5. Validation of MYC-LUAD subnetwork by ChIP-seq and ATAC-seq, and LUAD TF network by ATAC-seq. a, Overlap Venn diagram of MYC targets defined by SJARACNe inference from RNA-seq of TCGA-LUAD, by ChIP-seq in A549 cells (a commonly-used LUAD cell line), and by ATAC-seq in A549 cells. Narrow Peaks for A549 Chip-Seq of MYC were downloaded from ENCODE (ENCFF542GMN) and annotated to hg38 known gene region by ChIPseeker with default settings. A549 ATAC-seq results were downloaded from ENCODE (ENCFF143XED) and annotated to hg38 known gene region by ChIPseeker with default settings. Footprinting analysis was performed to define MYC targets from ATAC-seq data. **b**, Fisher's Exact Test was used to test the enrichment of MYC targets in LUAD predicted by SJARACNe with MYC targets by ChIP-seq, by ATAC-seq, or their overlaps, as well as the enrichment of MYC targets defined by ChIP-seq and ATAC-seq. **c,** SJARACNe-inferred subnetworks of all the 1,565 TFs are significantly overlapped with targets defined by ATAC-seq of A549 cells. For each of the 1,565 TFs, Fisher's Exact Test (one-sided) was used to test the enrichment of SJARACNe targets with ATAC-seq defined targets in A549 cells. FDR was used for p-value adjustment. Each dot represents one TF with the –log10(p-value) as X-axis and the odds ratio as Y-axis. All 1,565 TFs showed significant enrichment with a cutoff (red dashed lines) of adjusted P-value < 0.01 and odds ratio > 2.

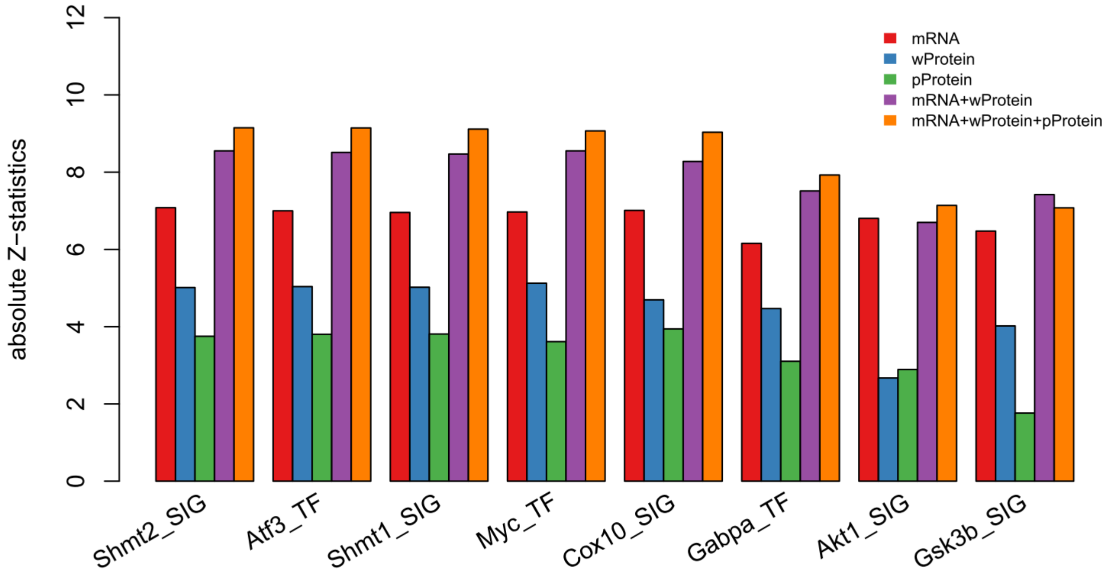

Figure S6. Comparison of different omics modalities for hidden driver inference in NetBID2. Bar plot for the absolute Z-statistics for the eight positive control drivers in Example 3

when using mRNA/transcriptomics only (red), wProtein/whole proteomics only (blue), pProtein/phosphoproteomics only (green), wRNA + wProtein (purple), and all three (orange).

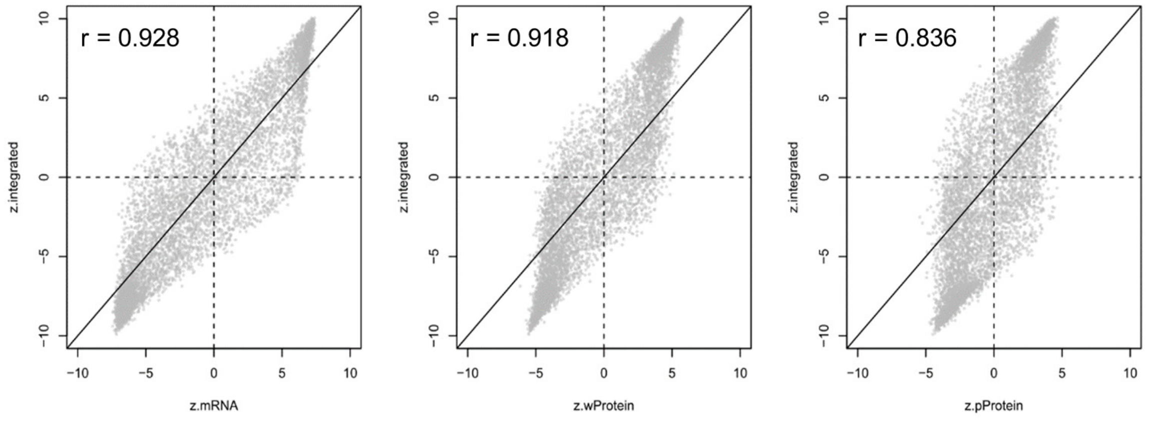

Figure S7. Correlation of Z-statistics between the integration of all three omics data and the usage of each omics modality. Each point represents one driver with its integrated Z-statistics (z.integrated) on the Y-axis and value from one omics on X-axis. Left to right: integrated vs. mRNA (transcriptomics) only, vs. wProtein (whole proteomics) only, and vs. pProtein (phosphoproteomics) only.

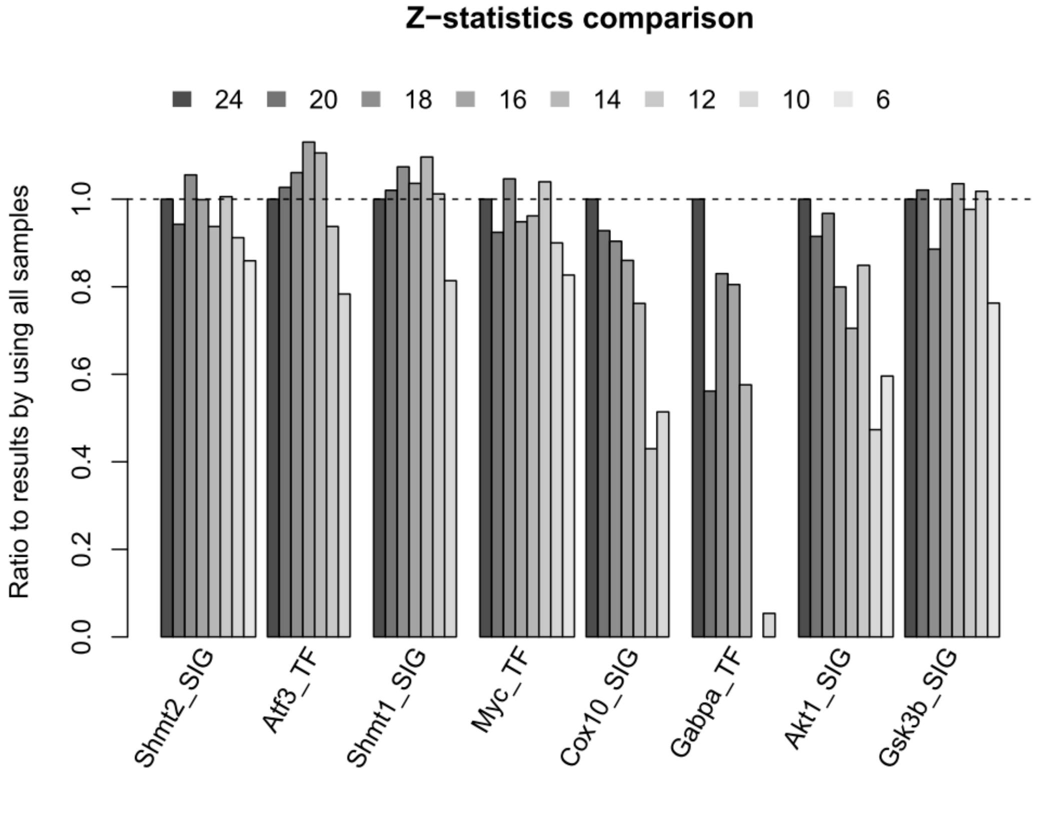

Figure S8. NetBID2 performance on the different numbers of samples. Bar plot for the ratio of Z-statistics for eight positive control drivers when using the different number of samples to generate the gene-regulatory network compared to the results using all 24 samples. For each driver, the number of samples to generate the network is 24, 20, 18,16, 14, 12, 10 to 6 from left to right.

Table S1. Summary of 145 pre-built context-specific transcription factor (TF) and signaling (SIG) networks in the NetBID2_Runner app. The summary is available at the following sites.
https://jyyulab.github.io/NetBID_shiny/docs/tutorial4online/GTEx_network
https://jyyulab.github.io/NetBID_shiny/docs/tutorial4online/TARGET_network
https://jyyulab.github.io/NetBID_shiny/docs/tutorial4online/TCGA_network

Dataset	Network File Prefix (hyperlinked to QC report)	Sample Size	No. of Hubs (TF)	No. of Hubs (SIG)	Total Genes	No. of Edges
GTEx	Adipose_Subcutaneous	763	1,898	8,482	18,350	748,106
GTEx	Adipose_Visceral_Omentum	564	1,880	8,407	19,971	527,263
GTEx	Adrenal_Gland	275	1,880	8,427	19,851	741,880
GTEx	Artery_Aorta	450	1,890	8,440	18,407	847,886
GTEx	Artery_Coronary	253	1,891	8,463	18,676	953,909
GTEx	Artery_Tibial	770	1,887	8,404	18,807	792,675
GTEx	Brain_Amygdala	177	1,898	8,395	21,345	577,441
GTEx	Brain_Anterior_cingulate_cortex_BA24	213	1,891	8,283	23,889	541,470
GTEx	Brain_Caudate_basal_ganglia	291	1,879	8,168	23,798	487,432
GTEx	Brain_Cerebellar_Hemisphere	263	1,899	8,421	21,557	549,276
GTEx	Brain_Cerebellum	298	1,896	8,453	20,728	559,565
GTEx	Brain_Cortex	325	1,901	8,421	22,555	564,814
GTEx	Brain_Frontal_Cortex_BA9	425	1,902	8,405	22,986	557,345
GTEx	Brain_Hippocampus	243	1,900	8,336	22,086	572,179
GTEx	Brain_Hypothalamus	236	1,916	8,412	22,439	561,057
GTEx	Brain_Nucleus_accumbens_basal_ganglia	277	1,887	8,194	23,321	511,001
GTEx	Brain_Putamen_basal_ganglia	232	1,881	8,096	23,705	463,706
GTEx	Brain_Spinal_cord_cervical_c_1	182	1,905	8,448	20,474	588,439
GTEx	Brain_Substantia_nigra	164	1,903	8,445	21,480	549,376
GTEx	Breast_Mammary_Tissue	480	1,902	8,440	20,676	570,403
GTEx	Cells_EBV_transformed_lymphocytes	192	1,881	8,478	17,368	970,419
GTEx	Cells_Transformed_fibroblasts	217	1,854	8,162	22,066	395,354
GTEx	Colon_Sigmoid	389	1,896	8,492	18,664	845,675
GTEx	Colon_Transverse	432	1,881	8,281	23,135	426,845
GTEx	Esophagus_Gastroesophageal_Junction	401	1,886	8,456	18,879	747,937
GTEx	Esophagus_Mucosa	622	1,878	8,409	20,043	523,176
GTEx	Esophagus_Muscularis	559	1,878	8,463	19,742	644,457
GTEx	Heart_Atrial_Appendage	452	1,868	8,343	20,116	549,378
GTEx	Heart_Left_Ventricle	689	1,833	8,005	24,012	402,611
GTEx	Liver	251	1,840	8,353	20,141	685,787
GTEx	Lung	867	1,895	8,559	20,448	674,020
GTEx	Minor_Salivary_Gland	181	1,894	8,522	18,981	706,291
GTEx	Muscle_Skeletal	1,132	1,871	8,253	19,592	455,219

GTEx	Nerve_Tibial	722	1,897	8,561	18,272	716,865
GTEx	Ovary	195	1,892	8,503	18,167	987,284
GTEx	Pancreas	360	1,859	8,316	20,349	465,621
GTEx	Pituitary	301	1,911	8,590	17,976	846,479
GTEx	Prostate	262	1,910	8,623	18,700	847,158
GTEx	Skin_Not_Sun_Exposed_Suprapubic	638	1,907	8,540	19,138	718,220
GTEx	Skin_Sun_Exposed_Lower_leg	849	1,905	8,543	19,062	630,414
GTEx	Small_Intestine_Terminal_Ileum	193	1,902	8,407	22,145	502,693
GTEx	Spleen	260	1,874	8,550	19,348	762,506
GTEx	Stomach	381	1,842	8,216	21,290	465,055
GTEx	Testis	406	1,956	9,028	22,728	598,255
GTEx	Thyroid	812	1,902	8,544	18,526	739,934
GTEx	Uterus	166	1,889	8,512	17,919	924,647
GTEx	Vagina	173	1,897	8,548	19,818	707,535
GTEx	Whole_Blood	3,288	1,773	7,869	22,389	307,150
TARGET	ALL_log2TP50M.185_all_21659_21659_185	185	1,643	6,247	16,443	830,213
TARGET	ALL_log2TP50M.185_D_21585_21585_126	126	1,639	6,277	15,607	899,561
TARGET	ALL_log2TP50M.185_ETV6-RUNX1_21783_21783_19	19	1,679	6,436	15,679	1,542,021
TARGET	ALL_log2TP50M.185_Hyperdiploid_21802_21802_22	22	1,650	6,294	14,674	977,288
TARGET	ALL_log2TP50M.185_NoneOfKnownSubType_21519_21519_88	88	1,644	6,263	15,433	797,609
TARGET	ALL_log2TP50M.185_R_21299_21299_59	59	1,639	6,199	15,046	696,241
TARGET	ALL_log2TP50M.185_TCF3.fusion_21489_21489_20	20	1,633	6,259	15,067	1,436,612
TARGET	ALL_log2TP50M.185_TrisomyChr4_10_21944_21944_19	19	1,651	6,433	15,344	1,173,781
TARGET	AML_log2TP50M.202_all_20615_20615_202	202	1,565	5,886	16,376	739,275
TARGET	AML_log2TP50M.202_D_20573_20573_157	157	1,557	5,856	16,396	738,591
TARGET	AML_log2TP50M.202_HighRisk_20606_20606_14	14	1,541	5,843	11,815	10,295,917
TARGET	AML_log2TP50M.202_Inv_16_20301_20301_34	34	1,483	5,616	17,104	573,949
TARGET	AML_log2TP50M.202_LowRisk_20414_20414_80	80	1,530	5,788	15,643	706,094
TARGET	AML_log2TP50M.202_M1_20624_20624_49	49	1,568	5,946	13,882	760,174
TARGET	AML_log2TP50M.202_M1_20772_20772_23	23	1,582	5,936	14,642	788,584
TARGET	AML_log2TP50M.202_M2_20624_20624_49	49	1,568	5,946	13,882	760,174
TARGET	AML_log2TP50M.202_M4_21055_21055_57	57	1,529	5,755	16,082	728,678

TARGET	AML_log2TP50M.202_M5_20649_20649_36	36	1,577	5,904	14,795	607,962
TARGET	AML_log2TP50M.202_MLL_20716_20716_39	39	1,576	5,912	14,706	580,075
TARGET	AML_log2TP50M.202_NormalCytogenetic_20682_20682_38	38	1,582	5,928	15,090	610,264
TARGET	AML_log2TP50M.202_OtherCytogenetic_20730_20730_46	46	1,581	5,955	14,295	697,323
TARGET	AML_log2TP50M.202_R_21425_21425_45	45	1,588	6,004	13,305	715,123
TARGET	AML_log2TP50M.202_StandardRisk_20772_20772_96	96	1,589	5,961	15,141	785,297
TARGET	AML_log2TP50M.202_t_8to21_21352_21352_28	28	1,557	5,916	14,062	711,192
TARGET	CCSK_log2TP50M.13_all_22209_22209_13	13	1,675	6,242	13,472	6,201,442
TARGET	NBL_log2TP50M.167_all_22031_22031_167	167	1,691	6,321	16,858	864,162
TARGET	NBL_log2TP50M.167_Diagnosis_22052_22052_158	158	1,691	6,318	16,866	914,270
TARGET	NBL_log2TP50M.167_MKI_High_22135_22135_35	35	1,684	6,286	15,764	641,363
TARGET	NBL_log2TP50M.167_MKI_Intermediate_23049_23049_48	48	1,702	6,398	14,956	776,178
TARGET	NBL_log2TP50M.167_MKI_Low_22479_22479_52	52	1,681	6,309	15,085	826,050
TARGET	NBL_log2TP50M.167_MYCN_Amp_22417_22417_36	36	1,691	6,292	15,004	654,906
TARGET	NBL_log2TP50M.167_MYCN_NonAmp_22979_22979_130	130	1,691	6,326	16,613	934,672
TARGET	OS_log2TP50M.76_all_25328_25328_76	76	1,513	5,929	14,388	1,125,398
TARGET	OS_log2TP50M.76_Metastatic_23065_23065_18	18	1,566	6,092	15,224	1,527,570
TARGET	OS_log2TP50M.76_NonMetastatic_25600_25600_57	57	1,529	6,048	14,055	905,869
TARGET	TALL_log2TP50M.261_all_27218_27218_261	261	1,653	6,271	16,514	1,068,228
TARGET	TALL_log2TP50M.261_LMO2_LYL1_26382_26382_18	18	1,653	6,271	14,983	1,923,168
TARGET	TALL_log2TP50M.261_TAL1_2_27164_27164_95	95	1,653	6,271	15,760	1,031,057
TARGET	TALL_log2TP50M.261_TAL1_27169_27169_87	87	1,653	6,271	15,552	1,032,180
TARGET	TALL_log2TP50M.261_TLX1_3_27189_27189_70	70	1,653	6,271	14,854	950,515
TARGET	TALL_log2TP50M.261_TLX1_3_HOXA_27202_27202_102	102	1,653	6,271	14,936	1,145,757
TARGET	TALL_log2TP50M.261_USP7.MU_26972_26972_33	33	1,653	6,271	15,608	776,229
TARGET	TALL_log2TP50M.261_USP7.WT_27220_27220_227	227	1,653	6,271	16,346	1,089,701
TARGET	WT_log2TP50M.124_all_22067_22067_124	124	1,692	6,309	14,287	1,188,700

TARGET	WT_log2TP50M.124_D_22064_22064_118	118	1,690	6,298	14,074	1,153,750
TARGET	WT_log2TP50M.124_DAWT_21818_21818_38	38	1,693	6,274	12,973	563,191
TARGET	WT_log2TP50M.124_FHWT_22093_22093_86	86	1,693	6,308	14,095	1,031,527
TARGET	WT_log2TP50M.124_StageI_21998_21998_16	16	1,690	6,324	16,137	2,111,918
TARGET	WT_log2TP50M.124_StageII_22037_22037_53	53	1,692	6,305	12,779	699,740
TARGET	WT_log2TP50M.124_StageIII_21969_21969_36	36	1,700	6,325	13,725	612,622
TARGET	WT_log2TP50M.124_StageIV_V_21965_21965_19	19	1,689	6,281	14,653	822,950
TCGA	ACC.T_33753_15879_77	77	1,634	6,049	15,766	2,678,153
TCGA	BLCA.T_34890_16552_348	348	1,654	6,152	17,456	1,153,515
TCGA	BRCA.N_35169_16784_109	109	1,650	6,231	16,773	1,410,937
TCGA	BRCA.T_35306_16556_1058	1,058	1,654	6,110	17,280	701,060
TCGA	CESC.T_34635_16448_241	241	1,651	6,130	16,724	1,495,925
TCGA	CHOL.T_34535_16625_35	35	1,642	6,113	15,006	595,519
TCGA	COADREAD.N_34500_16548_50	50	1,638	6,174	15,968	965,465
TCGA	COADREAD.T_34941_16029_568	568	1,640	5,887	17,155	568,255
TCGA	DLBC.T_34146_15754_45	45	1,639	5,978	16,610	610,360
TCGA	ESCA.T_36407_17732_70	70	1,693	6,373	13,954	1,728,755
TCGA	GBM.T_35189_16907_116	116	1,675	6,191	14,562	2,081,008
TCGA	HNSC.N_34287_16805_41	41	1,654	6,133	16,533	952,649
TCGA	HNSC.T_35077_16623_477	477	1,657	6,117	17,589	993,816
TCGA	KICH.N_34695_16995_25	25	1,654	6,194	15,167	836,699
TCGA	KICH.T_34369_16307_66	66	1,623	6,091	15,863	1,218,200
TCGA	KIRC.N_34806_16840_72	72	1,642	6,216	17,990	1,291,690
TCGA	KIRC.T_35436_16605_512	512	1,646	6,108	18,155	725,352
TCGA	KIRP.N_34657_16795_32	32	1,637	6,162	18,352	740,083
TCGA	KIRP.T_34670_16163_281	281	1,621	6,033	19,217	780,254
TCGA	LAML.T_33701_16232_130	130	1,563	5,951	14,151	2,011,399
TCGA	LGG.T_35181_16688_505	505	1,666	6,093	18,727	798,548
TCGA	LIHC.N_32688_15698_48	48	1,551	5,933	14,137	918,499
TCGA	LIHC.T_34138_16137_255	255	1,610	6,080	17,020	1,162,974
TCGA	LUAD.N_34391_16621_58	58	1,618	6,133	16,758	1,213,383
TCGA	LUAD.T_35321_16788_493	493	1,661	6,198	16,199	1,069,901
TCGA	LUSC.N_35008_16941_47	47	1,637	6,205	15,267	1,034,058
TCGA	LUSC.T_35519_17032_498	498	1,674	6,246	16,935	1,511,084
TCGA	MESO.T_34729_16696_82	82	1,664	6,139	14,888	1,750,719
TCGA	OV.T_35775_17075_403	403	1,666	6,243	16,271	1,171,973
TCGA	PAAD.T_35229_16763_176	176	1,661	6,252	18,242	1,299,578
TCGA	PCPG.T_34539_16173_170	170	1,653	6,072	17,710	1,047,047
TCGA	PRAD.N_34860_16629_52	52	1,662	6,207	15,888	1,037,788

TCGA	PRAD.T_34895_16552_469	469	1,653	5,983	20,390	565,336
TCGA	SARC.T_35040_16566_249	249	1,665	6,139	17,110	1,358,015
TCGA	SKCM.M_34930_16593_355	355	1,656	6,133	17,777	1,537,624
TCGA	SKCM.T_34695_16581_96	96	1,653	6,125	16,059	1,617,019
TCGA	STAD.N_35194_16921_22	22	1,654	6,214	15,620	2,686,623
TCGA	STAD.T_36385_17413_247	247	1,682	6,344	16,069	1,826,747
TCGA	TGCT.T_36008_17274_144	144	1,716	6,389	17,629	1,208,686
TCGA	THCA.N_34727_16599_57	57	1,635	6,158	15,904	1,135,046
TCGA	THCA.T_34492_16400_489	489	1,619	5,954	19,664	648,107
TCGA	THYM.T_34883_16290_111	111	1,652	6,151	19,648	857,301
TCGA	UCEC.N_35024_16794_35	35	1,632	6,108	15,371	760,476
TCGA	UCEC.T_35791_16480_529	529	1,675	6,126	17,753	822,108
TCGA	UCS.T_35280_16963_55	55	1,695	6,281	15,031	1,022,125
TCGA	UVM.T_33177_15751_76	76	1,612	5,892	16,350	1,144,831

Table S2. Literature supports the eight positive controls in Example 3.

Genes	Change direction	Experimental validation	Reference	PubMed PMID
Cox10	up	COX10 is crucial for T-cell activation in vitro and in vivo	Tan et al., Immunity 2017	28285833
Shmt1	up	Enzymes of One-Carbon Metabolism are Induced In Vivo during T Cell activation	Ron-Harel et al., Cell Metabolism 2016	27411012
Shmt2	up	SHMT2 Is Critical for Mitochondrial One-Carbon Metabolism and T Cell Survival in vitro and in vivo	Ron-Harel et al., Cell Metabolism 2016	27411012
Myc	up	Myc controls metabolic reprogramming upon T lymphocyte activation in vitro and in vivo	Wang et al., Immunity 2011	22195744
ATF3	up	ATF3 regulates signaling pathways involved in immuno-responsiveness	Ku et al., Front. Endocrinol. 2020	32922364
GABPA	up	GABP is essential for the regulation of IL-7Rα expression in T cells in vitro and in vivo	Xue et al., Nat. Immu. 2004	15361867
AKT1	up	TcRζ/CD3 and TcRζ/CD3-CD28 signaling require the guanine nucleotide exchange factor (GEF) Vav-1 as well as the activation of phosphatidylinositol 3-kinase, protein kinase B (PKB/AKT), and its inactivation of glycogen synthase kinase-3 (GSK-3).	Wood et al., JBC 2006	16905544
GSK3B	Down			
